# In the Wnt of Paneth Cells: Immune-Epithelial Crosstalk in Small Intestinal Crohn’s Disease

**DOI:** 10.3389/fimmu.2017.01204

**Published:** 2017-09-26

**Authors:** Nicole S. Armbruster, Eduard F. Stange, Jan Wehkamp

**Affiliations:** ^1^Internal Medicine I, University Hospital Tübingen, Tübingen, Germany

**Keywords:** Paneth cell, Crohn’s disease, defensins, Wnt signaling, monocytes

## Abstract

Paneth cells, specialized secretory epithelial cells of the small intestine, play a pivotal role in host defense and regulation of microbiota by producing antimicrobial peptides especially—but not only—the human α-defensin 5 (HD5) and HD6. In small intestinal Crohn’s disease (CD) which is an entity of inflammatory bowel diseases, the expression of HD5 and HD6 is specifically compromised leading to a disturbed barrier and change in the microbial community. Different genetically driven but also non-genetic defects associated with small intestinal CD affect different lines of antimicrobial Paneth cell functions. In this review, we focus on the mechanisms and the crosstalk of Paneth cells and bone marrow-derived cells and highlight recent studies about the role of the Wnt signaling pathway in this connection of ileal CD. In summary, different lines of investigations led by us but also now numerous other groups support and reconfirm the proposed classification of this disease entity as Paneth’s disease.

## Introduction

In approximately 70% of Crohn’s disease (CD) patients, the small intestine is affected, the remainder have colonic disease only (1). Mostly due to refluxing colonic contents, the small intestine also harbors large populations of microbes with a complex repertoire of various bacterial species, although their density is much lower than in the colon by about 3 orders of magnitude (2). Specializing in the production of different antimicrobial peptides (AMPs), Paneth cells are responsible for the host defense in this part of the intestinal tract. Generally, AMPs are the first line of defense of the human body. These innate immune effector molecules serve as endogenous antibiotics protecting the host against the multitude of commensals and pathogens with their antimicrobial activity. In humans and mammals, AMPs consist of various protein families consisting of two major families: the defensins and cathelicidins. They are small cationic peptides with amphipathic characteristics (3). Human defensins are characterized by their beta-sheet structure and are subdivided in the two main groups of α- and β-defensins by the structure of the six disulfide-connected cysteines (4). Different defensins and AMPs are expressed by all barrier-epithelial cell tissues throughout the entire body but they are also found in circulating immune cells playing an important role in host defense (e.g., most abundant content of neutrophils). While human β-defensins are produced by all epithelial surfaces (including the skin, gastrointestinal, respiratory, and urogenital tract), human α-defensin 5 (HD5) and HD6 are expressed dominantly by small intestinal-epithelial-secretory Paneth cells (5, 6).

In this review, we focus in particular on the pivotal role of the Wnt signaling pathway in the immune-epithelial crosstalk in small intestinal CD. Reflecting the complexity of gut homeostasis, epithelial Paneth cells are closely linked to bone marrow-derived cells and monocytes are directly controlling Paneth cells *via* Wnt signaling. Besides ileal CD, this is likely also relevant for graft-versus-host diseases.

## Paneth Cells

Paneth cells are specialized secretory epithelial cells located in the small intestine on the bottom of the crypts of Lieberkühn. They originate from crypt stem cells and are filled with secretory granules containing large quantities of antimicrobial proteins and peptides. Besides the human α-defensins HD5 and HD6, these include lysozyme, regenerating islet-derived 3 gamma (Reg3γ), and secretory phospholipase A_2_. Taken together, Paneth cells play an important role in the maintenance of the intestinal barrier function (6, 7). The expression levels of the α-defensins HD5 and HD6 are about three to one and exceeds those of other peptides including lysozyme or phospholipase A_2_ up to 100-fold (8, 9). The α-defensins HD5 and HD6 are constitutively expressed with varying levels in different diseases, whereas the Reg3γ production by Paneth cells is induced in the presence of microbes in the intestinal lumen (10, 11). Furthermore, Paneth cells have an influence on the microbial composition of the small intestine. With their expression of various antimicrobials, they protect the intestine from pathogens and limit the number of commensals in the crypts (6). In addition, Paneth cells are implicated in stem cell regulation (1). The expression of the pattern-recognition receptor nucleotide-binding oligomerization domain containing 2 (NOD2) and its activation secures stem cell survival essential for tissue regeneration and healing processes (12).

## Crohn’s Disease

Inflammatory bowel disease (IBD) is a chronic inflammation of the gastrointestinal tract characterized by an infiltration of various immune cells as a result of a pathological interaction of the commensal microbiota within the mucosa. IBD is classified in ulcerative colitis (UC) and CD. UC is restricted to the colon and typically shows continuous mucosal inflammation, whereas CD potentially arises all along the gastrointestinal tract characterized by a patchy discontinuous inflammation (13). Depending on the localization of the lesions, CD is subdivided into ileum only (L1), colon only (L2), or both ileum and colon (L3) (14), and this phenotype of CD location is remarkably stable over time (15).

### CD—Imbalance of Microbiota and Innate and Adaptive Immune Response

During the relapsing course of their disease, CD patients predominantly suffer from abdominal pain and diarrhea (14). In the healthy gut, immune homeostasis prevails with a gut microbiota that is in balance with intestinal epithelial cells producing AMPs and releasing immune modulatory cytokines that drive naïve dendritic cells (DCs) to differentiate into tolerogenic DCs that trigger the priming of regulatory T cells. In contrast when an imbalance exists between an under-protected mucosa and an altered, usually less diversified microbiota naïve DCs may differentiate into immunogenic DCs initiating the priming of effector T cells leading to inflammation (16).

### Link of Ileal CD and Paneth Cells: “Paneth’s Disease”

It has been shown that in ileal CD a reduced expression of mucosal AMPs leads to inflammation and an attenuated antimicrobial defense by the mucosa (17). In a mouse model of CD-like ileitis, Schaubeck et al. showed a loss of the Paneth cell product lysozyme (18). Patients with ileal CD showed a decreased constitutive expression of the α-defensins HD5 and HD6 produced by Paneth cells. The reduction of HD5 and HD6 was further associated with mutations in the NOD2 receptor (10). It was the first evidence pointing toward the Paneth cell when Lala et al. discovered that in the intestinal mucosa not only macrophages but also Paneth cells express high levels of NOD2 (19). Several mutations in the NOD2 gene were detected, so that this intracellular receptor for bacterial muramyl-dipeptide was the first susceptibility gene in ileal CD and about 30% of patients who suffer from ileal CD carry this mutation (20).

However, CD is related to many other genetic defects that also lead to impaired Paneth cell function (1) and to Paneth cell necroptosis (21). Further examples are the autophagy gene autophagy-related 16-like 1 (Atg16L1) that plays an important role in CD pathogenesis by affecting Paneth cell granule exocytosis in patients with an ileal phenotype (22) or the transcription factor X-box binding protein 1 (XBP1) of the endoplasmic reticulum stress response activated during an inflammation. XBP1 defect leads to Paneth cell disturbance and increased susceptibility to IBD (23). In the IBD linkage region on chromosome 19q13, the calcium-mediated potassium channel subfamily N member 4 (KCNN4) is situated. The KCNN4 encoded protein plays a pivotal role in Paneth cell secretion and showed reduced expression levels in NOD2-mutated ileal CD patients (24). Furthermore, in the mucosa of ileal CD patients, adherent bacteria are present (25), probably caused by compromised antimicrobials Paneth cell function. Consequently, a compromised antibacterial defense due to reduced α-defensin expression or secretion explains many features of ileal CD, even though the mechanisms are complex and vary in term of pathways and origin.

## Wnt Signaling in Ileal CD

**Figure 1:**
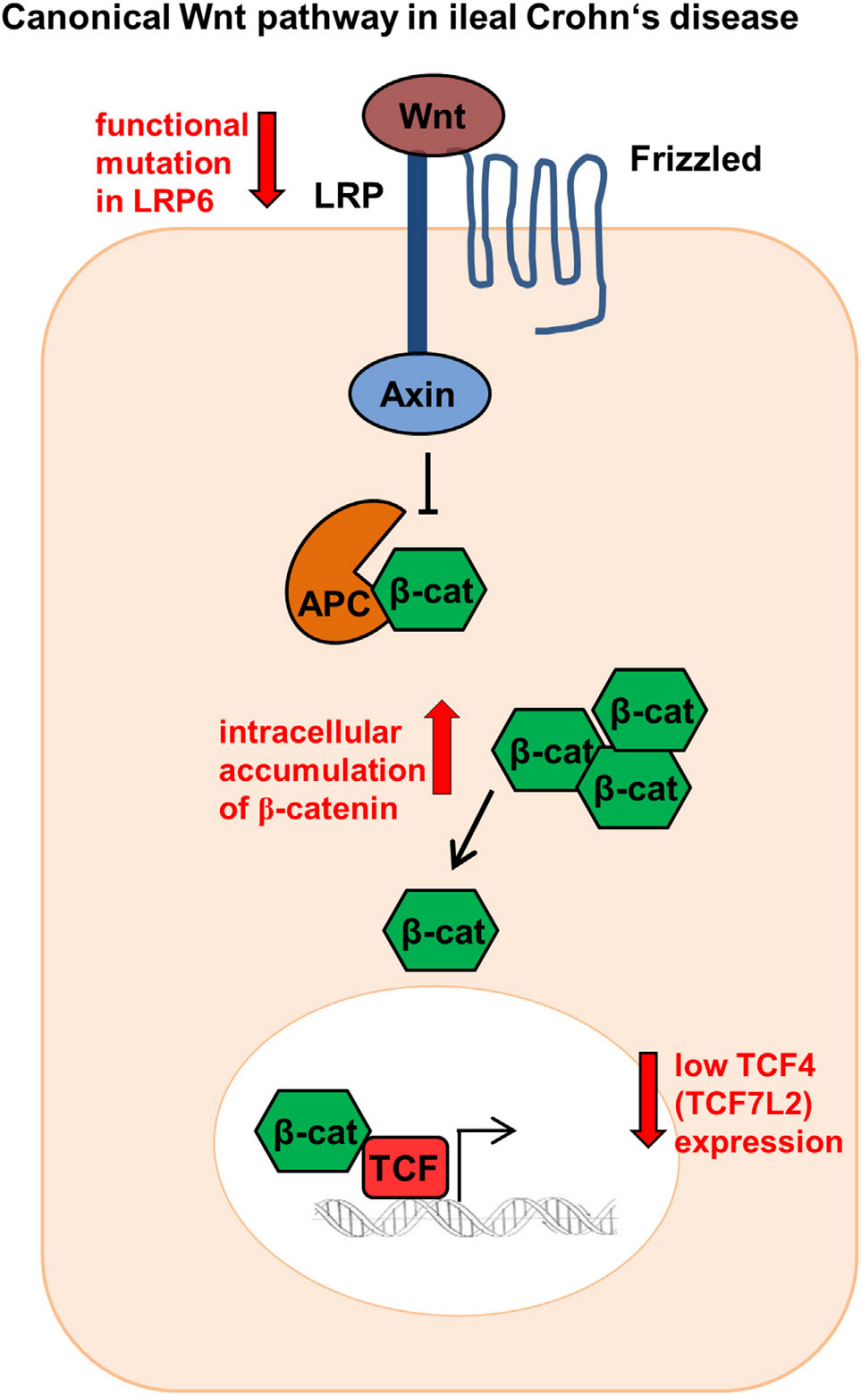
Canonical Wnt signaling in ileal Crohn’s disease (CD). Patients show reduced TCF-4 and TCF-1 expression as well as modifications in the co-receptor lipoprotein receptor-related protein 6 (LRP6). In addition, ileal CD patients indicate a β-catenin accumulation intracellularly.

The Wnt signaling pathway plays a pivotal role in the gut mucosal homeostasis and therefore in the intestinal epithelium. Wnt is an important element keeping intestinal epithelial stem cells in a proliferating status, enabling stem cell maintenance (26), and provoking the differentiation and maturation process of Paneth cells, thereby regulating the expression of the alpha-defensins HD5 and HD6 (27). The canonical Wnt pathway is activated when Wnt ligands released from epithelial cells, Paneth cells, or mesenchymal cells bind to the cell surface receptor “Frizzled” (28). They ultimately mediate the stabilization of β-catenin that can then transfer into the nucleus. Nuclear β-catenin binds to the transcription factors, T-cell factor 4 (TCF-4 or TCF7L2) and lymphoid enhancer factor, and enables target gene transcription such as the antibacterial defensin genes alpha 5 and 6 (*DEFA5* and *DEFA6*), the genes for HD5 and HD6 (29) (Figure 1).

Previously our group could show that in ileal CD there is a link between the reduced expression of Paneth cell α-defensins HD5 and HD6 and the Wnt transcription factor TCF-4 (TCF7L2). Ileal CD patients showed diminished TCF-4 expression irrespective of the inflammation status (25). However, not only TCF-4 is involved, the expression levels of the Wnt signaling effector TCF-1 were also reduced in ileal CD patients (30). Furthermore, the co-receptor low-density lipoprotein receptor-related protein 6 (LRP6), a further Wnt factor playing a key role in the cytoplasmic stabilization of β-catenin, was also modified in CD leading to diminished HD5 expression (31). Ileal CD patients diagnosed under the age of 18 showed a 10.63% higher mutation rate for the single-nucleotide polymorphism LRP6 rs2302685 (31). This turns LRP6 into an appealing therapeutic target toward early onset ileal CD. Not only variations of co-receptors or transcription factors are impaired in CD. A further study demonstrated that both β-catenin and E-cadherin accumulate intracellularly in an unusual fashion and showed an altered localization in the plasma membrane in CD patients (32) (Figure 1).

**Figure 2:**
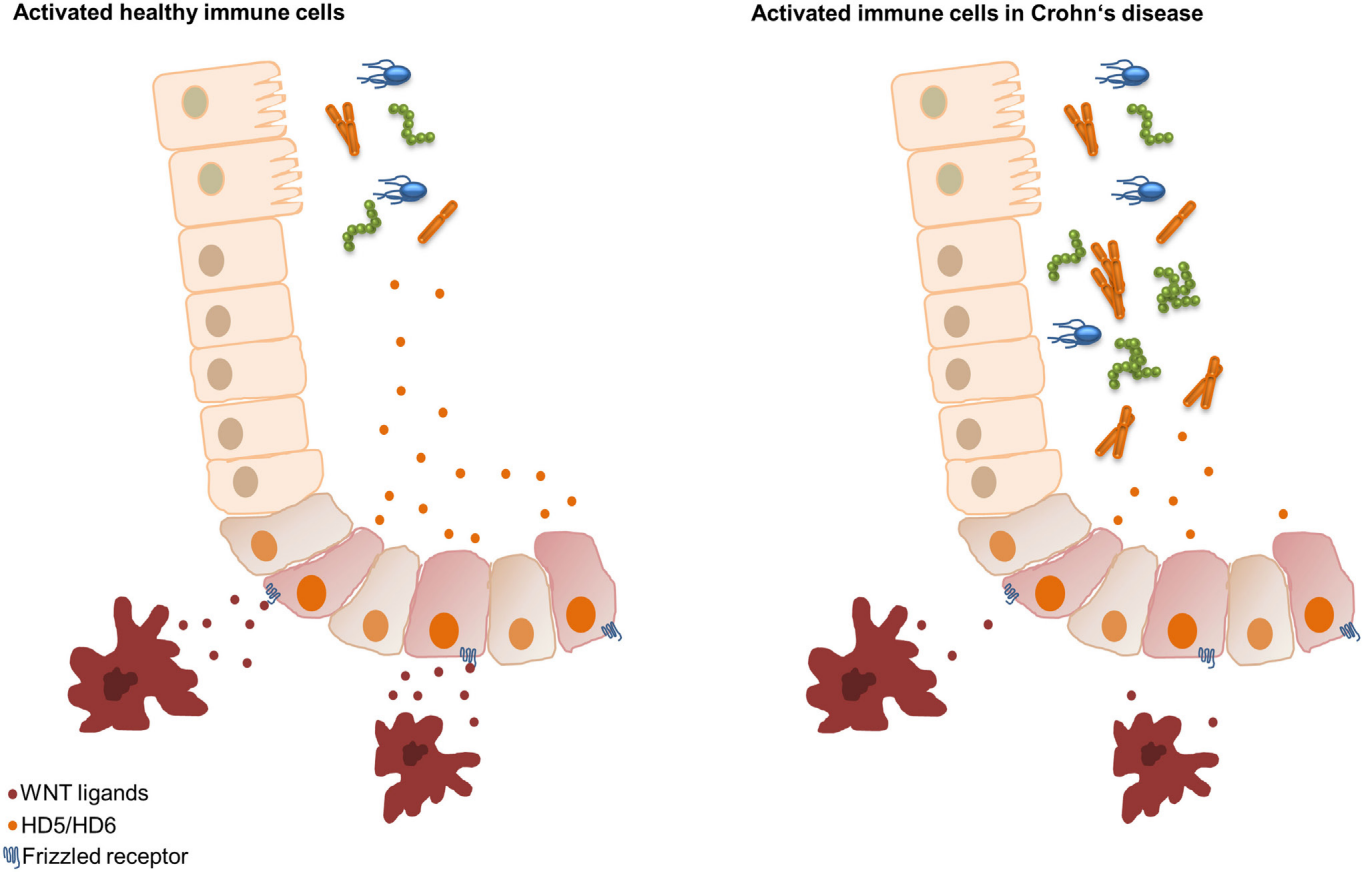
Hypothesis of the Wnt signaling Paneth cell connection in ileal Crohn’s disease (CD). In healthy controls, classical monocytes produce Wnt ligands leading to an inducible upregulation of human α-defensin 5 (HD5) and HD6. Classical monocytes of patients with ileal CD show reduced Wnt ligand expression, resulting in an impaired inducibility of HD5 and HD6, leading to bacterial invasion.

In addition, we recently found that peripheral blood mononuclear cells (PBMCs) of healthy controls reconstituted the decreased HD5 and HD6 levels of ileal CD patients. We demonstrated that the driving force of the PBMC effect was the Wnt ligand expression and not the cytokine release. The monocytes of CD patients expressed significantly lower values of the canonical Wnt ligands Wnt3, Wnt3a, Wnt1, and the wntless Wnt ligand secretion mediator (33). At the same time, measured cytokines did not show significant differences. So, a further very essential mechanism in CD is the connection between Paneth cells and bone marrow-derived monocytes characterized by an attenuated intestinal barrier function through a reduced Wnt ligand expression in PBMCs. Therefore, we hypothesize that in ileal inflammation circulating immune cells, probably classical monocytes as the main subset (3), supply the necessary Wnt ligands that lead to the production of HD5 and HD6 in Paneth cells resulting in an enhanced intestinal barrier function. However, in ileal CD, the monocytes show reduced Wnt ligand expression, thereby negatively affecting the secretion of the AMPs HD5 and HD6 and leading to bacterial infiltration and chronic inflammation (33) (Figure 2). But, the potential mechanisms of reduced Wnt delivery from monocytes still remain unknown.

## Conclusion

Here, in this review, we emphasized the important role of ileal defensins of the Paneth cell in host defense mechanisms of the small intestine. Our group could show that in ileal CD patients, the defective barrier leads to an invasion of different bacteria around the mucosa. This is a direct consequence of the various Paneth cell defects leading to a reduced α-defensin expression in ileal CD. However, we recently found out that the infiltrating monocytes in ileal CD patients showed a compromised Wnt ligand production leading to an impaired defensin-inducing capacity. In conclusion, there is a defect interaction between Paneth cells and monocytes in ileal CD but the exact mechanisms of the regulation of Wnt ligand expression in monocytes have to be investigated to further develop new therapeutic strategies in intestinal disorders.

## Author Contributions

NA, ES, and JW wrote and discussed the manuscript.

## Conflict of Interest Statement

The authors declare that the research was conducted in the absence of any commercial or financial relationships that could be construed as a potential conflict of interest. The reviewer MS and handling editor declared their shared affiliation.

## References

[1] Wehkamp J Stange EF Paneth’s disease. J Crohns Colitis (2010) 4:523–31. 10.1016/j.crohns.2010.05.010 21122555

[2] Eckburg PB Bik EM Bernstein CN Purdom E Dethlefsen L Sargent M Diversity of the human intestinal microbial flora. Science (2005) 308:1635–8. 10.1126/science.1110591 15831718 PMC1395357

[3] Zasloff M. Antimicrobial peptides of multicellular organisms. Nature (2002) 415:389–95. 10.1038/415389a 11807545

[4] Hill CP Yee J Selsted ME Eisenberg D. Crystal structure of defensin HNP-3, an amphiphilic dimer: mechanisms of membrane permeabilization. Science (1991) 251:1481–5. 10.1126/science.2006422 2006422

[5] Tunzi CR Harper PA Bar-Oz B Valore EV Semple JL Watson-MacDonell J Beta-defensin expression in human mammary gland epithelia. Pediatr Res (2000) 48:30–5. 10.1203/00006450-200007000-00008 10879797

[6] Ouellette AJ Bevins CL Paneth cell defensins and innate immunity of the small bowel. Inflamm Bowel Dis (2001) 7:43–50. 10.1097/00054725-200102000-00007 11233660

[7] Ostaff MJ Stange EF Wehkamp J. Antimicrobial peptides and gut microbiota in homeostasis and pathology. EMBO Mol Med (2013) 5:1465–83. 10.1002/emmm.201201773 24039130 PMC3799574

[8] Wehkamp J Chu H Shen B Feathers RW Kays RJ Lee SK Paneth cell antimicrobial peptides: topographical distribution and quantification in human gastrointestinal tissues. FEBS Lett (2006) 580:5344–50. 10.1016/j.febslet.2006.08.083 16989824

[9] Bevins CL. The Paneth cell and the innate immune response. Curr Opin Gastroenterol (2004) 20:572–80. 10.1097/00001574-200411000-00012 15703685

[10] Wehkamp J Harder J Weichenthal M Schwab M Schäffeler E Schlee M NOD2 (CARD15) mutations in Crohn’s disease are associated with diminished mucosal alpha-defensin expression. Gut (2004) 53:1658–64. 10.1136/gut.2003.032805 15479689 PMC1774270

[11] Wehkamp J Salzman NH Porter E Nuding S Weichenthal M Petras RE Reduced Paneth cell alpha-defensins in ileal Crohn’s disease. Proc Natl Acad Sci U S A (2005) 102:18129–34. 10.1073/pnas.0505256102 16330776 PMC1306791

[12] Nigro G Rossi R Commere P-H Jay P Sansonetti PJ. The cytosolic bacterial peptidoglycan sensor Nod2 affords stem cell protection and links microbes to gut epithelial regeneration. Cell Host Microbe (2014) 15:792–8. 10.1016/j.chom.2014.05.003 24882705

[13] Xavier RJ Podolsky DK. Unravelling the pathogenesis of inflammatory bowel disease. Nature (2007) 448:427–34. 10.1038/nature06005 17653185

[14] Podolsky DK Inflammatory bowel disease. N Engl J Med (2002) 347:417–29. 10.1056/NEJMra020831 12167685

[15] Louis E Collard A Oger AF Degroote E Aboul Nasr El Yafi FA Belaiche J Behaviour of Crohn’s disease according to the Vienna classification: changing pattern over the course of the disease. Gut (2001) 49:777–82. 10.1136/gut.49.6.777 11709511 PMC1728556

[16] Maynard CL Elson CO Hatton RD Weaver CT. Reciprocal interactions of the intestinal microbiota and immune system. Nature (2012) 489:231–41. 10.1038/nature11551 22972296 PMC4492337

[17] Wehkamp J Koslowski M Wang G Stange EF Barrier dysfunction due to distinct defensin deficiencies in small intestinal and colonic Crohn’s disease. Mucosal Immunol (2008) 1:S67–74. 10.1038/mi.2008.48 19079235

[18] Schaubeck M Clavel T Calasan J Lagkouvardos I Haange SB Jehmlich N Dysbiotic gut microbiota causes transmissible Crohn’s disease-like ileitis independent of failure in antimicrobial defence. Gut (2016) 65:225–37. 10.1136/gutjnl-2015-309333 25887379 PMC4752651

[19] Lala S Ogura Y Osborne C Hor SY Bromfield A Davies S Crohn’s disease and the NOD2 gene: a role for Paneth cells. Gastroenterology (2003) 125:47–57. 10.1016/S0016-5085(03)00661-9 12851870

[20] Ogura Y Bonen DK Inohara N Nicolae DL Chen FF Ramos R A frameshift mutation in NOD2 associated with susceptibility to Crohn’s disease. Nature (2001) 411:603–6. 10.1038/35079114 11385577

[21] Günther C Martini E Wittkopf N Amann K Weigmann B Neumann H Caspase-8 regulates TNF-α-induced epithelial necroptosis and terminal ileitis. Nature (2011) 477:335–44. 10.1038/nature10400 21921917 PMC3373730

[22] Cadwell K Liu JY Brown SL Miyoshi H Loh J Lennerz JK A key role for autophagy and the autophagy gene Atg16l1 in mouse and human intestinal Paneth cells. Nature (2008) 456:259–63. 10.1038/nature07416 18849966 PMC2695978

[23] Kaser A Lee AH Franke A Glickman JN Zeissig S Tilg H XBP1 links ER stress to intestinal inflammation and confers genetic risk for human inflammatory bowel disease. Cell (2008) 134:743–56. 10.1016/j.cell.2008.07.021 18775308 PMC2586148

[24] Simms LA Doecke JD Roberts RL Fowler EV Zhao ZZ McGuckin MA KCNN4 gene variant is associated with ileal Crohn’s disease in the Australian and New Zealand population. Am J Gastroenterol (2010) 105:2209–17. 10.1038/ajg.2010.161 20407432

[25] Wehkamp J Wang G Kübler I The Paneth cell α-defensin deficiency of ileal Crohn’s disease is linked to Wnt/Tcf-4. J Immunol (2007) 179(5):3109–18. 10.4049/jimmunol.179.5.3109 17709525

[26] Schuijers J Clevers H. Adult mammalian stem cells: the role of Wnt, Lgr5 and R-spondins. EMBO J (2012) 31:2685–96. 10.1038/emboj.2012.149 22617424 PMC3380219

[27] van Es JH Jay P Gregorieff A van Gijn ME Jonkheer S Hatzis P Wnt signalling induces maturation of Paneth cells in intestinal crypts. Nat Cell Biol (2005) 7:381–6. 10.1038/ncb1240 15778706

[28] Farin HF Van Es JH Clevers H. Redundant sources of Wnt regulate intestinal stem cells and promote formation of Paneth cells. Gastroenterology (2012) 143:1518–29.e7. 10.1053/j.gastro.2012.08.031 22922422

[29] Logan CY Nusse R. The Wnt signaling pathway in development and disease. Annu Rev Cell Dev Biol (2004) 20:781–810. 10.1146/annurev.cellbio.20.010403.113126 15473860

[30] Beisner J Teltschik Z Ostaff MJ Tiemessen MM Staal FJ Wang G TCF-1-mediated Wnt signaling regulates Paneth cell innate immune defense effectors HD-5 and -6: implications for Crohn’s disease. Am J Physiol Gastrointest Liver Physiol (2014) 307:G487–98. 10.1152/ajpgi.00347.2013 24994854

[31] Koslowski MJ Kübler I Chamaillard M Schaeffeler E Reinisch W Wang G Genetic variants of Wnt transcription factor TCF-4 (TCF7L2) putative promoter region are associated with small intestinal Crohn’s disease. PLoS One (2009) 4:e4496 10.1371/journal.pone.0004496 19221600 PMC2637978

[32] Muise AM Walters TD Glowacka WK Griffiths AM Ngan BY Lan H Polymorphisms in E-cadherin (CDH1) result in a mis-localised cytoplasmic protein that is associated with Crohn’s disease. Gut (2009) 58:1121–7. 10.1136/gut.2008.175117 19398441

[33] Courth LF Ostaff MJ Mailänder-Sánchez D Malek NP Stange EF Wehkamp J Crohn’s disease-derived monocytes fail to induce Paneth cell defensins. Proc Natl Acad Sci U S A (2015) 112:14000–5. 10.1073/pnas.1510084112 26512113 PMC4653149

